# Bedside zero-fluoroscopy temporary permanent pacemaker implantation in the electrical storm treatment under the guidance of EnSite 3D system: a case report

**DOI:** 10.1186/s13019-024-02579-0

**Published:** 2024-02-06

**Authors:** Lei Wang, Pengfei Li, Jun li, Jicheng Xi, Zhibiao Zhang, Fang Yan, Yanan Zhang, Haixiong Wang, Huiyuan Han

**Affiliations:** 1 Department of Cardiology, Shanxi Cardiovascular Hospital, 18 Yifen Road, Taiyuan, Shanxi 030001 China; 2 Department of Cardiology, Xinzhou People’s Hospital, Xinzhou, Shanxi 034000 China; 3 Department of Cardiac Surgery, Shanxi Cardiovascular Hospital, Taiyuan, Shanxi 030001 China; 4 Department of Critical Medicine, Shanxi Bethune Hospital, Taiyuan, Shanxi 030001 China

**Keywords:** Temporary pacing, Electrical storm, Navigation system

## Abstract

**Background:**

Electrical storm (ES) is a clinical emergency characterized by multiple malignant ventricular arrhythmias or ICD discharges within 24 h, requiring early rational management.

**Case presentation:**

We report a 55-year-old man who underwent aortic valve replacement experienced recurrent ventricular tachycardia/ventricular fibrillation. A temporary permanent pacemaker with the EnSite system was implanted, and significant inhibition of the electrical storm, attributed to the atrial overdrive pacing, ensued.

**Conclusions:**

In emergency regarding an electrical storm, the bedside temporary permanent pacemaker implantation with the EnSite system is concluded to be feasible and safe.

## Background

Electrical storm (ES) is a common type of arrhythmia in critically severe patients requiring urgent electrical cardioversion or defibrillation [1]. However, simple sedation and antiarrhythmic therapy are, in some cases, unsuccessful, especially with patients experiencing ventricular tachycardia (VT)/ventricular fibrillation (VF) induced by ventricular premature. In this context, traditional temporary pacemaker implantation is recommended as a viable solution for increasing the patients’ heart rates, thereby inducing ventricular overdrive pacing to suppress VT/VF [2]. However, for many patients, the therapeutic effect will be affected under the following conditions [3]: (1) Dislodgment of the temporary electrode, which will affect overdrive suppression; (2) Temporary right ventricular apical pacing with a frequency > 90 per min more, leading to loss of atrial function and synchrony between left and right ventricles, in turn severely affecting the cardiac function in patients and thus inducing heart failure [4]; (3) Requirement of multiple life-support equipment (ECMO, ventilator, bedside hemofilter, and infusion pump) limits patient activity, consequently causing failure of implantation under fluoroscopy in the catheterization lab. In addition, bedside floating temporary electrodes can only be applied to the right ventricle (with effect on cardiac function) with a potential risk of electrode dislodgement/dislocation. In the face of these deficiencies, bedside temporary permanent AAI/DDD pacemaker implantation with the 3D navigation system becomes a viable alternative.

## Case presentation

**Fig. 1:**
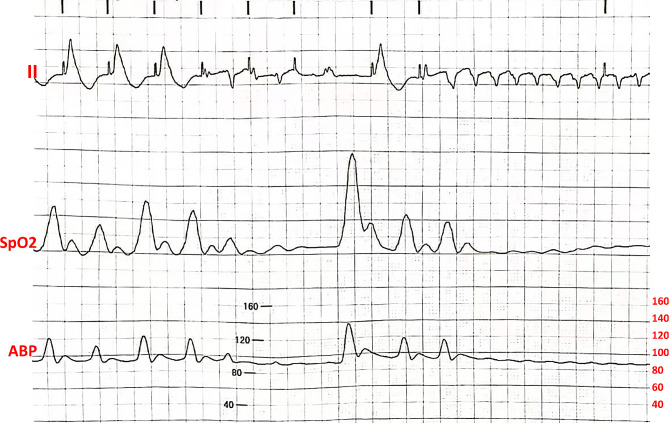
Electrocardiogram of the tachycardia with temporary overdrive pacing placed in the septeum of right ventricle

Due to severe aortic stenosis, a 55-year-old man underwent aortic valve replacement with cardiopulmonary bypass under hypothermia and general anesthesia. Postoperatively, weaning was difficult; therefore, a ventricular assist device ECMO was employed. However, the patient had recurrent VT/VF due to the ventricular premature, considering an electrical storm. Deep sedation and antiarrhythmic treatment with Amiodarone and Esmolol were ineffective. After administering local anesthesia, we obtained venous access from the left subclavian vein. A bedside floating electrode was then inserted into the right ventricle and right ventricular high-septal pacing (HSP) was displayed on the electrocardiogram (ECG). A frequency of 110 per min failed to achieve overdrive suppression for the ventricular premature with episodes of the electrical storm (Fig. 1). A frequency of 120 per min was found to be effective; however, echocardiography revealed reduced ventricular wall motion with significantly low invasive arterial blood pressure. Meanwhile, an intermittent pacemaker malfunction occurred, inducing subsequent attacks of VT/VF. After discussion, it was decided that active lead implantation should be performed into the right atrial appendage with the EnSite 3D system, along with ventricular active electrode implantation if necessary. Briefly, an Inquiry (Abbott, USA) electrophysiology catheter was introduced through the left subclavian vein and was used to reconstruct the right atrium, tricuspid annulus, right ventricle, and superior vena cava (SVC). His-bundle area and the right appendage mapping were also performed.

**Fig. 2:**
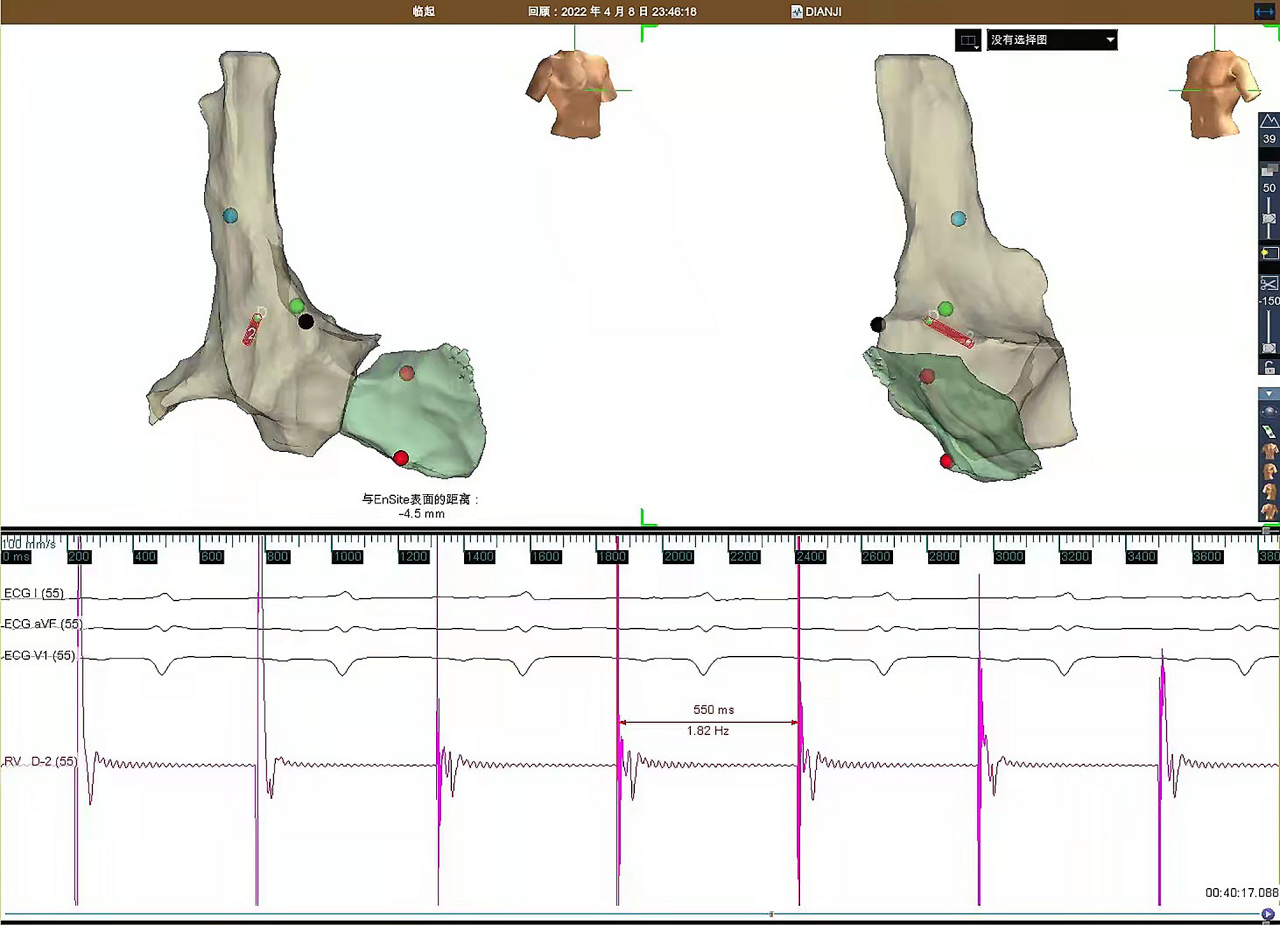
EnSite image showing the site of the successful lead implanation in the right atrium using the right anterior oblique (left) left anterior oblique (right) views

The pacing electrode (Medtronic, 5076-52) was inserted into the right atrium (RA) via the left subclavian vein, and its tail was connected to the EnSite 3D system using an alligator cable. Under the guidance system, showing the 3D tracking catheter and lead movement in the vein and cardiac chamber models, we adjusted the lead to the central part of the RA. Subsequently, a J-type atrial Stylet was inserted, and the head end of the atrial active electrode was sent to the right atrial appendage (RAA). High-amplitude A waves are displayed as a result. The electrode was then proceeded forward, and the Stylet was removed. Tests were conducted for the sensing, pacing threshold, current of injury (COI), and impedance. It was found that 1:1 atrioventricular conduction was achieved under atrial pacing with a frequency of 110 per min (Fig. 2).

**Fig. 3:**
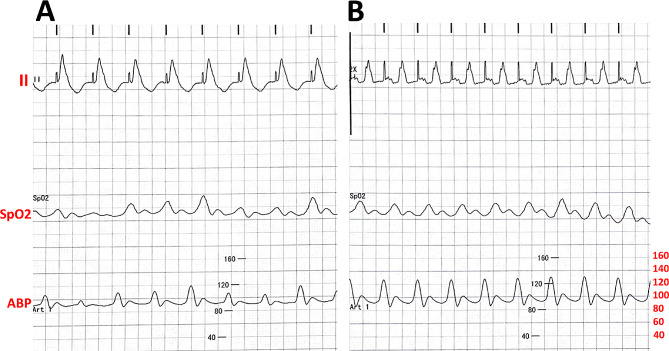
Electrocardiogram, SpO2 and arterial pressure profiles in temporary ventricle pacing (**A**) and temporary Permanent atrial pacing (**B**)

Considering that the atrioventricular nodal conduction was sufficient for VT suppression, AAI pacing at 110 per minute was performed. The lead was fixed and connected to the temporary pacemaker. Under atrial pacing at 110 per min, the invasive blood pressure (Fig. 3) and cardiac ejection fraction (EF) on ultrasound were significantly improved compared to those under ventricular pacing.

## Discussion and conclusions

Temporary permanent pacemakers are commonly used as a bridge therapy after pacemaker removal due to local pocket/systemic infection before reimplantation, or in the context of unstable hemodynamics requiring urgent pacing [5]. It is superior to using conventional temporary pacemakers due to a lower rate of electrode dislodgement, infection, and embolization, given the use of an active fixation lead implanted through the internal jugular/subclavian veins [6]. Many studies have reported the effectiveness of X-ray guided temporary permanent pacemaker implantation in the transitional therapy of patients [7, 8]. However, temporary permanent pacemaker implantation under the guidance of a zero-fluoroscopy 3D mapping system has not yet been reported.

In the current patient, a conventional temporary pacemaker was initially applied with a balloon-tipped catheter used to float the pacing leads into the right ventricle at an effective pacing frequency of 120 per min. Cardiac ultrasound revealed impaired ventricular wall motion, while the blood pressure was kept at 115/88 mmHg, and intermittent pacemaker dysfunction led to episodes of VF/VT. The above-mentioned phenomena suggested potential electrode dislodgement and adverse effects of right ventricular pacing on heart function. Therefore, we did consider the possibility of re-implanting overdrive atrial pacing with an active fixation tip. Given that the patient was in a sedated state and had limited movement due to the application of multiple advanced life-support equipment (e.g., ECMO, and ventilator), active atrial lead implantation with the EnSite system was performed to induce high-frequency atrial pacing.

Compared with other mapping systems, such as Carto and Localisa, the EnSite system is more affordable, has greater accuracy in cardiac chamber reconstruction (especially in the right atria and appendage), and is safer to use due to the tip of the inserted electrode being visible even when separate from the created matrix [9]. Previous studies demonstrated zero-fluoroscopy approach implantations of permanent pacemakers or cardiac resynchronization therapy device using Ensite system are safe and feasible [10, 11]. Here, we used the Inquiry (Abbott, USA) catheter to reconstruct the SVC, right atria, and partial right ventricle, accurately mapped the HIS and right appendage, and visualized the pacing lead with the EnSite system. Upon reaching the right appendage, the electrode position was adjusted according to the potential and was then followed by testing for sensing amplitude, pacing threshold, pacing impedance, and COI. Subsequently, 1:1 atrioventricular conduction was achieved under atrial pacing with a frequency of 110 per min, sufficient for VT suppression.

An electrical storm is a clinical emergency requiring early management. Beta-blockers and amiodarone are preferred agents for this issue. Administration of other anti-arrhythmic drugs (AADs), such as lidocaine, and catheter ablation of triggering premature ventricular complexes (PVCs), are other potential therapies for electrical storm. In guidelines from The European Society of Cardiology, overdrive pacing therapy was recently recommended as a viable solution in patients with ventricular arrhythmia who poorly respond to pharmaceutical therapy [12]. A low heart rate leads to heterogeneity of ventricular repolarization, and the subsequent ventricular premature stimuli induce reentry and VF/VT in the setting of this tissue heterogeneity. The overdrive pacing method is capable of decreasing the incidence of ventricular premature. In the meantime, it can increase the likelihood of the ventricular premature entering the absolute refractory period of the myocardial tissue, thereby suppressing the incidence of VT [13].

In the present case, we also compared the ventricular wall motion via cardiac ultrasound and the invasive blood pressure upon atrial and ventricular pacing with a frequency of 110 per min. We observed that atrial pacing contributed to improved wall motion and blood pressure, indicating the superiority of AAI pacing in suppressing VT. In patients with intact VA conduction (> AV conduction), ventricular pacing can disrupt AV synchrony and unfavorably affect hemodynamics, triggering symptoms of pacemaker syndrome [14]. Additionally, it also highlighted the significance of the synchrony between left and right ventricles in improving cardiac function.

It is feasible to perform bedside temporary permanent pacing lead implantation with the EnSite system for patients suffering from the electric storm. Nevertheless, large-scale studies are required to further validate its safety and effectiveness.

## Data Availability

The datasets supporting the conclusions of this article are included within the article.

## Abbreviations

ES: Electrical storm
VT: ventricular tachycardia
VF: ventricular fibrillation
ECMO: Extracorporeal Membrane Oxygenation
HSP: high-septal pacing
ECG: electrocardiogram
SVC: superior vena cava
RAA: right atrial appendage
RA: right atrium
EF: ejection fraction
COI: current of injury
